# Quantitative Ultrasound Texture Analysis of Breast Tumors: A Comparison of a Cart-Based and a Wireless Ultrasound Scanner

**DOI:** 10.3390/jimaging11050146

**Published:** 2025-05-06

**Authors:** David Alberico, Lakshmanan Sannachi, Maria Lourdes Anzola Pena, Joyce Yip, Laurentius O. Osapoetra, Schontal Halstead, Daniel DiCenzo, Sonal Gandhi, Frances Wright, Michael Oelze, Gregory J. Czarnota

**Affiliations:** 1 Physical Sciences, Sunnybrook Research Institute, Toronto, ON M4N 0A4, Canada; david.alberico@sunnybrook.ca (D.A.); lakshmanan.sannachi@sunnybrook.ca (L.S.); marialourdes.anzolapena@sunnybrook.ca (M.L.A.P.); waiszejoyce.yip@sunnybrook.ca (J.Y.); laurentiusoscar.osapoetra@sunnybrook.ca (L.O.O.);; 2 Division of Medical Oncology, Department of Medicine, Sunnybrook Health Sciences Centre, Toronto, ON M4N 0A4, Canada; sonal.gandhi@sunnybrook.ca; 3 Department of Medicine, University of Toronto, Toronto, ON M5S 1A1, Canada; 4 Department of Surgical Oncology, Department of Surgery, Sunnybrook Health Sciences Centre, Toronto, ON M4N 0A4, Canada; frances.wight@sunnybrook.ca; 5 Department of Surgery, University of Toronto, Toronto, ON M5S 1A1, Canada; 6 Department of Electrical and Computer Engineering, University of Illinois Urbana-Champaign, Champaign, IL 61801, USA; oelze@illinois.edu; 7 Department of Radiation Oncology, Sunnybrook Health Sciences Centre, Toronto, ON M4N 0A4, Canada; 8 Department of Medical Biophysics, University of Toronto, Toronto, ON M5S 1A1, Canada; 9 Department of Radiation Oncology, University of Toronto, Toronto, ON M5S 1A1, Canada

**Keywords:** breast cancer, quantitative ultrasound, texture analysis, wireless ultrasound

## Abstract

Previous work has demonstrated quantitative ultrasound (QUS) analysis techniques for extracting features and texture features from ultrasound radiofrequency data which can be used to distinguish between benign and malignant breast masses. It is desirable that there be good agreement between estimates of such features acquired using different ultrasound devices. Handheld ultrasound imaging systems are of particular interest as they are compact, relatively inexpensive, and highly portable. This study investigated the agreement between QUS parameters and texture features estimated from clinical ultrasound images of breast tumors acquired using two different ultrasound scanners: a traditional cart-based system and a wireless handheld ultrasound system. The 28 patients who participated were divided into two groups (benign and malignant). The reference phantom technique was used to produce functional estimates of the normalized power spectra and backscatter coefficient for each image. Root mean square differences of feature estimates were calculated for each cohort to quantify the level of feature variation attributable to tissue heterogeneity and differences in system imaging parameters. Cross-system statistical testing using the Mann–Whitney U test was performed on benign and malignant patient cohorts to assess the level of feature estimate agreement between systems, and the Bland–Altman method was employed to assess feature sets for systematic bias introduced by differences in imaging method. The range of *p*-values was 1.03 × 10^−4^ to 0.827 for the benign cohort and 3.03 × 10^−10^ to 0.958 for the malignant cohort. For both cohorts, all five of the primary QUS features (MBF, SS, SI, ASD, AAC) were found to be in agreement at the 5% confidence level. A total of 13 of the 20 QUS texture features (65%) were determined to exhibit statistically significant differences in the sample medians of estimates between systems at the 5% confidence level, with the remaining 7 texture features being in agreement. The results showed a comparable magnitude of feature variation between tissue heterogeneity and system effects, as well as a moderate level of statistical agreement between feature sets.

## 1. Introduction

Ultrasound waves propagating through a tissue undergo scattering, with some of the wave energy being backscattered toward the transducer and used to produce an image based on the measured signal intensity versus depth. The strength of the signal depends on the acoustic scattering properties of the tissue being imaged, and the spectral properties of the backscattered signal contain information about the scattering properties of the tissue. Quantitative ultrasound (QUS) is a body of methods for obtaining quantitative measures of tissue properties from ultrasound radiofrequency (RF) data. Signal processing techniques performed on a segment of the RF signal corresponding to the tissue region of interest are used to produce estimates of the normalized power spectrum (NPS) of the backscattered ultrasound signal as well as the tissue backscatter coefficient (BSC). Features of these estimated spectra can then be used to make inferences about tissue microstructure. Ultrasound scanners typically used for clinical imaging applications can also serve as data collection tools for QUS methods if they allow the user to collect post-beamforming RF data.

QUS techniques are used in a wide variety of tissue characterization applications, such as distinguishing between benign and malignant breast masses [1,2]. Texture-based analysis methods applied to 2D parametric maps of QUS feature measures have demonstrated a correlation between changes in QUS feature and texture feature measures and changes in tissue microstructure related to cell death [3,4]. Multi-feature classification models for predicting the response of locally advanced breast cancer (LABC) to neoadjuvant chemotherapy at an early stage of the treatment regimen have been developed from QUS feature and texture feature measures using machine learning techniques, with some models demonstrating prediction accuracies above 80% [5,6]. 

The potential for QUS techniques to serve as valuable tools for breast cancer diagnosis and response monitoring has been repeatedly demonstrated, but in order for these techniques to see widespread adoption in clinical practice, it is necessary to demonstrate that they are reproducible using different ultrasound systems and transducers. One way to establish this is to conduct detailed comparisons of the estimates of power spectra, backscatter coefficients, and QUS features made using image data collected with different ultrasound devices. Previous work has demonstrated an agreement in QUS feature estimates for tissue mimicking phantoms with known acoustic properties made using different ultrasound systems [7,8,9]. In 2010, Wirtzfeld et al. investigated the reproducibility of backscatter coefficient measurements of rat tumors, imaging six animal models using three clinical ultrasound systems and one laboratory system with a total of nine different ultrasound transducers, and reported reasonable agreement between systems [10]. This was followed by another study published in 2015 comparing BSC estimates produced by three different systems for tumors grown from two different breast cancer cell lines, which again found good agreement between BSC estimates made using different ultrasound systems [11]. More recently, a comparative investigation of the QUS feature estimates of human breast tumors produced using two different clinical ultrasound systems was performed [12]. In that study, the ultrasound systems being investigated were both traditional cart-based imaging systems. 

Interest in hand-held portable ultrasound systems has been increasing in recent years [13,14,15]. The small size and portability of portable ultrasound systems offer clear advantages over bulky cart-based systems, and their role in clinical practice can be expected to grow as they become more widely available and affordable. The main limitation of portable ultrasound compared to traditional cart-based systems is the typically poorer image resolution. Since adoption of portable ultrasound in a clinical setting is a relatively recent phenomenon, there has been little investigation into the suitability of handheld ultrasound systems for performing QUS feature estimation in a clinical context. As the use of both portable ultrasound and QUS-based techniques in clinical practice continues to grow, the importance of this line of investigation will also increase. 

This study aimed to investigate the level of agreement between QUS feature measures produced using a traditional cart-based clinical ultrasound system and a handheld portable system. Specifically, the systems under consideration were the Sonix Touch (ST) (Ultrasonix Medical Corporation, Richmond, BC, Canada) and the Clarius L15HD (CL15) portable ultrasound scanner (Clarius Mobile Health, Vancouver, BC, Canada). The Clarius L15HD is a portable linear-array scanner which retails for CAD 4190 at the time of writing that allows the operator to export the raw RF data necessary for performing QUS feature estimation. The Sonix Touch was selected for comparison because QUS feature estimates obtained from ultrasound RF data captured using this system have been used for characterizing benign and malignant breast lesions in previous work by Osapoetra et al. [1].

QUS feature determination was performed using a clinical cohort of 28 women presenting with unidentified breast masses suspected of malignancy which were imaged using both ultrasound systems. The level of agreement in QUS feature and texture feature estimates for both benign and malignant sub-groups was quantified in order to assess the suitability of the QUS feature estimation techniques used in previous work for use with Clarius L15HD image data.

## 2. Materials and Methods

### 2.1. Ultrasound Systems and Scanning Protocol

Ultrasound RF data were acquired using two different clinical ultrasound systems: a cart-based Sonix Touch, developed by Ultrasonix Medical Corp. (Richmond, BC, Canada), and the wireless handheld Clarius L15HD system from Clarius Mobile Health (Vancouver, BC, Canada). For the Sonix Touch, an L14-5/60 linear array transducer operating at a transmit–receive frequency of 10 MHz was used to collect image data. This system and transducer were selected as this same configuration was used for data collection in a previous study investigating QUS feature estimates of benign and malignant breast masses [1]. The CL15 uses an in-built linear-array transducer which operates at 10 MHz when the imaging depth is set to 4 cm or greater. The 6 dB bandwidth and center frequency of the Sonix Touch L14-5/60 and the Clarius L15 transducer were measured using a plane reflector method [16,17]. Relevant properties of the two transducers are summarized in Table 1.

**Figure 1:**
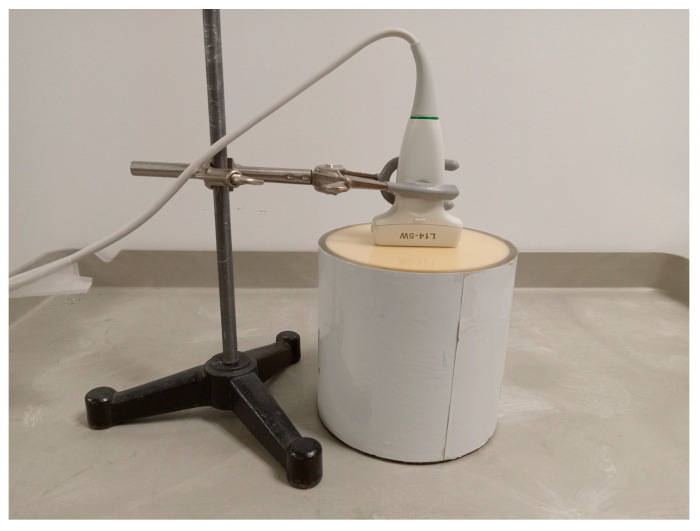
Apparatus used for imaging of ultrasound reference phantom.

For phantom measurements, ultrasound data were acquired for both systems as follows. The transducer was fixed in a stand and aligned perpendicular to the phantom’s scanning window. The stand and the phantom were placed on a flat surface and the transducer was then lowered until it was in contact with the scanning window. Acoustic coupling was established through the application of ultrasound gel. This configuration is shown in Figure 1.

Ultrasound images of patient breast masses were acquired before any clinical intervention. Eligibility to participate in this study required only that the patient be presenting with suspected breast cancer and have the ability and willingness to consent to a breast ultrasound scan. Since this study was concerned with breast masses before any clinical intervention, the recruitment criteria excluded patients who had received chemotherapy, radiotherapy, or major breast surgery within a 4-week period prior to a proposed scan. Women with breast implants were also excluded from consideration for the study. In total, 28 women presenting with unidentified breast masses suspected of malignancy at the Rapid Diagnostic Unit (RDU) of the Louise Temerty Breast Cancer Centre (Sunnybrook Health Sciences Center, Toronto, ON, Canada) consented to participate in the study. Suspicion of malignancy was determined by RDU staff; patients were approached for recruitment prior to additional diagnostic imaging or biopsy procedures.

Collection of clinical breast images was conducted as follows. With each ultrasound system, a pair of reference images along the tumor’s longest dimension and in the perpendicular imaging plane were taken, followed by a panning scan across the tumor volume with the transducer oriented in the radial direction with respect to the nipple. Focal depth and transducer transmit–receive frequencies remained consistent across patients. Scan procedures and technique were kept consistent for both ultrasound systems.

### 2.2. Ultrasound Data Analysis

Between 4 and 6 frames of ultrasound RF data were selected from each patient scan for QUS analysis. Image frames were selected to be free of any significant ultrasound artefacts and were spaced out along the width of the tumor volume in order to be representative of the tumor as a whole. Ultrasound readers selected a complex polygonal ROI around the tumor boundary by hand while referring to available clinical images of the tumor to aid with selection. Custom software and a graphical user interface created using MATLAB (The MathWorks Inc. (Natick, MA, USA) MATLAB Version: 9.1.0.1012177 (R2016b). Available at https://www.mathworks.com. Accessed on 1 January 2025. Custom code available from the authors upon request.) were used to facilitate ROI segmentation. ROI selections were confirmed by a participating radiologist with over 10 years of breast ultrasound research experience.

**Figure 2:**
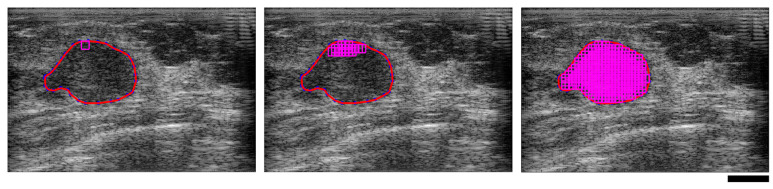
Ultrasound B-mode images of a malignant breast mass illustrating region of interest (ROI) selection, depicting a single window (**left**), several overlapped windows (**centre**), and the fully segmented ROI (**right**). The red line is the tumor boundary selected to define the limits of the ROI. The magenta squares are the overlapped windowed data blocks. The black scale bar represents 1 cm.

QUS feature determination was performed for all clinical images as follows. Each ROI was subdivided into discrete square data blocks 2 mm in dimension (roughly 10λ by 10λ, where λ is the ultrasound wavelength at the transducer’s center frequency), created with a 94% overlap in both lateral and axial dimensions. An example B-mode image with a superimposed segmented ROI is shown in Figure 2. For each data block, the average power spectral density was calculated by applying a Hanning window before taking the square magnitude of the fast Fourier transform (FFT) of the signal and then averaging over all RF lines within the block. The resulting backscattered power spectrum was normalized using the reference phantom technique [18] with ultrasound image data collected from a homogenous reference phantom. 

The phantom used was composed of glass beads with diameters 5–30 μm embedded in a substrate of gelatin filled with suspended microscopic oil droplets which formed a homogenous scattering background. This phantom was created by the Medical Physics Department at the University of Wisconsin, Madison, WI, USA. Measurements of the phantom’s acoustic properties were performed at the Department of Electrical and Computer Engineering, University of Illinois, Urbana, IL, USA. Attenuation and speed of sound were measured using the insertion loss and arrival time difference techniques, respectively [19,20]. The attenuation coefficient of the phantom was 0.7861 dB/(MHz-cm), and the speed of sound was 1488 m/s. The phantom’s backscatter coefficient was measured using a plane reflector method [16,17]. Four spherically focused transducers with center frequencies of 1.5, 3.5, 7.5, and 13 MHz were employed to perform the backscatter co-efficient measurement for the frequency band 1.5–20 MHz. 

In order to correct for the effects of attenuation, both total and local attenuation correction techniques were employed. For total attenuation, a breast tissue attenuation coefficient of 1 dB/(MHz-cm) was assumed based on the literature for tissue acoustic properties [21]. For local attenuation within the tumor volume itself, a reference-phantom based method was used [22]. First, the average normalized power spectrum at each axial depth within the ROI was computed at each frequency within the bandwidth of interest. Linear curve fitting to the results plotted against the axial depth within the tumor was then performed and the slope of the resulting linear fit was used to estimate the attenuation coefficient. The attenuation was then divided by frequency for the system’s frequency bandwidth and averaged. Attenuation correction was applied to each data block individually according to its depth below the tissue surface.

The set of QUS features to be investigated were chosen based on previous work demonstrating their relevance for characterizing benign and malignant breast masses [1]. The mid-band fit (MBF), spectral slope (SS), and spectral intercept (SI) were estimated by performing linear regression analysis of the log-compressed normalized power spectrum over the transducer 6 dB frequency bandwidth following attenuation compensation and spectrum normalization. Average scatterer diameter (ASD) and average acoustic concentration (AAC) were obtained from estimates of the BSC, calculated from the normalized power spectrum as follows:

$$\sigma_{s}\left(f,z\right)=\frac{W_{s}\left(f,z\right)}{W_{r}\left(f,z\right)}\sigma_{r}(f)e^{4\left(\alpha_{m}\left(f\right)z_{m}+\alpha_{i}\left(f\right)z_{i}-\alpha_{r}\left(f\right)z\right)}$$

where z is the axial depth of the window block being analyzed with respect to the transducer ($z=z_{i}+z_{m})$; $z_{i}$ is the axial depth of the ROI defining the tissue-tumor interface (the distance relevant for total attenuation correction); $z_{m}$ is the axial depth of the window block with respect to the ROI (the distance relevant for local attenuation correction within the tumor volume itself); f is the frequency in MHz; $W_{s}\left(f,z\right)$ and $W_{r}\left(f,z\right)$ are the backscattered power spectra of the sample and the reference phantom, respectively; $\sigma_{s}\left(f,z\right)$ and $\sigma_{r}(f)$ are the backscatter coefficients of the sample and the reference phantom; and $\alpha_{m}(f)$, $\alpha_{m}(f)$, and $\alpha_{r}(f)$ are the attenuation coefficients for the tumor, intervening tissue, and reference phantom. The estimated sample BSC was then compared to the theoretical BSC ($\sigma_{theory}$) using a least-squares method in order to determine the value of scatterer diameter that minimized the least-squares difference between the two. The calculation of the theoretical BSC assumed a Gaussian form factor for the scatterers [23,24], given by the following:

$$\sigma_{theory}\left(f\right)=\frac{\pi^{4}}{36c^{4}}f^{4}a_{eff}^{6}\bar{n}\gamma_{0}^{2}{FF}_{G}(f,a_{eff})$$

$${FF}_{G}\left(f,a_{eff}\right)=e^{-0.827(\frac{2\pi f}{c}a_{eff})}$$

where ${FF}_{G}\left(f,a_{eff}\right)$ is the Gaussian form factor; $a_{eff}$ is the effective average scatterer size; and c is the speed of sound in breast tissue, taken here to be 1540 m/s as per measurements made using ultrasound tomography [25]. $\bar{n}$ is the average number of scatterers per unit volume and $\gamma_{0}^{2}$ is the mean square variation in the acoustic impedance between the scatterers and the surrounding medium, which taken together are called the acoustic concentration.

Feature estimates were obtained for each data block within the ROI and then averaged over the entire region to obtain estimates for the five ‘primary’ QUS features. The process was repeated for each frame; average feature estimates for each frame were averaged across frames to obtain a final set of feature estimates representative of the tumor volume. In addition to these mean QUS feature estimates, parametric maps for each of the five primary features were created by averaging estimates from overlapping data blocks at each pixel position on the original image.

A set of texture features were derived for each of these parametric maps using the gray-level co-occurrence matrix (GLCM) method [26]. Following methods employed in previous investigations [1,12], a total of 16 GLCMs were constructed for each parametric map by considering the four possible angular relations between neighboring pixels (0°, 45°, 90°, 135°) in combination with four different step sizes between compared pixels (1, 2, 3, and 4 pixels). Four texture features were computed for each GLCM and averaged: contrast (CON), correlation (COR), homogeneity (HOM), and energy (ENE). In total, a set of 25 features (5 mean QUS features and 20 texture features) were extracted from the ultrasound RF data.

Previous work [10,11] has demonstrated that when comparing feature estimates made using different ultrasound systems and transducers, special consideration must be made regarding the bandwidth used for performing QUS feature estimation and its relation to the bandwidths of the individual transducers. To investigate the impact of analysis bandwidth, the previously outlined process was performed using the region of overlap between the transducers’ 6 dB bandwidths (5.1–8.3 MHz, see Table 1), which happens to be synonymous with the L15′s 6 dB bandwidth. A second set of feature estimates was produced for the ST data using its 6 dB bandwidth of 3–8 MHz.

### 2.3. Consistency Analysis

To investigate the relative effects of tissue heterogeneity and ultrasound system characteristics on the QUS and texture parameter estimates for the cart-based and wireless handheld ultrasound systems, a comparative study of feature estimates was conducted for both a homogeneous reference phantom and a cohort of five breast tumors. To obtain a quantitative metric for estimating the effect of heterogeneity on feature estimates made by a single system, the root mean square differences (RMSD) between individual feature estimates from 5 separate frames of RF data and the mean were calculated for each feature:

$${RMSD}_{f}=\sqrt{\frac{\sum_{i=1}^{N}{\left(\hat{Q}-Q_{i}\right)}^{2}}{N}}i$$

where $\hat{Q}$ is the mean feature averaged across all frames, $Q_{i}$ is the feature for frame i, and N is the number of frames.

For a metric to quantify the impact of ultrasound system properties on feature estimates, the root mean square differences between averaged feature sets (RMSDUSs) extracted from data acquired using the two ultrasound systems were calculated for each feature by averaging the result for each tumor:

$${RMSD}_{US}=\sqrt{\frac{\sum_{i=1}^{n}{\left({\hat{Q}}_{ULXi}-{\hat{Q}}_{L15i}\right)}^{2}}{n}}$$

where ${\hat{Q}}_{ULXi}$ and ${\hat{Q}}_{L15i}$ are the averaged features estimated from the ST and L15 ultrasound data, respectively, and n is the number of samples (tumors).

### 2.4. Statistical Analysis

In order to evaluate the overall level of feature estimate agreement between the two systems for the clinical data, statistical testing was performed for each primary QUS feature (mid-band fit, spectral slope, spectral intercept, average scatterer diameter, and average acoustic concentration) and each texture feature (contrast, correlation, homogeneity, and energy). The patients were divided into two groups, one consisting of the patients with benign breast masses and the other consisting of those with malignant masses, and the individual feature estimates made for each of these groups were compared across ultrasound systems. Following previous work [12], the nonparametric Mann–Whitney U test, which tests the hypothesis that two sets of samples are drawn from the same population, was used to test for statistical agreement and compute associated *p*-values for each of the 25 features. Statistical analysis was performed using custom MATLAB code.

In addition, the Bland–Altman method was employed [27]. This is a graphical method for assessing the level of agreement between different methods of clinical measurement. Two sets of measurements acquired using different methodologies or devices are used to produce a plot of the average of each pair of measurements versus the difference between each pair of measurements. The limits of agreement are set at ±1.96 times the standard deviation, and the mean difference is calculated in order to graphically assess the level of agreement between measurement methods. For the present study, the measurements were the sets of QUS feature estimates produced for each tumor in the patient group, and the measurement methods being compared were the two ultrasound systems.

## 3. Results

**Figure 3:**
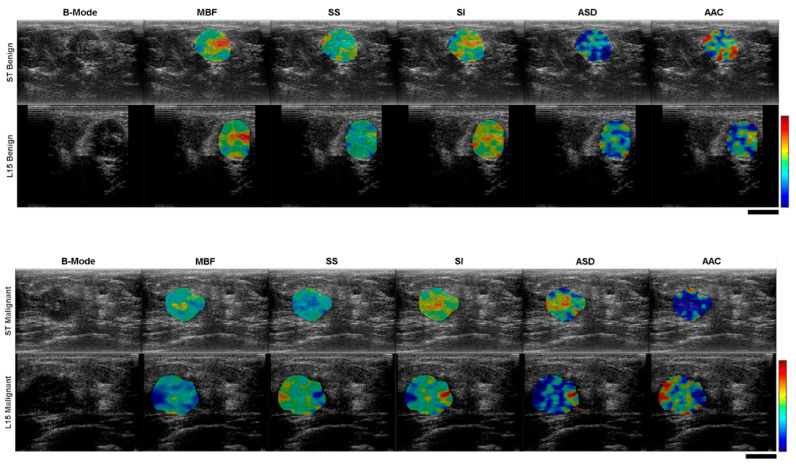
Cross-system comparison of ultrasound B-mode images with overlaid QUS feature parametric maps for representative benign (**top**) and malignant (**bottom**) clinical cases. The colour scale shows variation in feature magnitude. The black scale bars represent 1 cm.

The 28 patients recruited to participate in the study were divided into two cohorts; breast tissue biopsies performed as a part of normal clinical investigation by Rapid Diagnostics Unit staff determined that 22 of the breast lesions were malignant while the remaining 6 were classified as benign. Patient information is summarized in Table 2, including tumor grade, classification, and hormone receptor status: estrogen receptor (ER), progesterone receptor (PR), and human epidermal growth factor receptor 2 (HER2). For receptor status, a plus sign indicates positive status and a minus sign indicates negative status. Receptor status was not reported for benign cases and has been marked as N/A. The patients were between 35 and 97 years old with a median age of 51 years. The breast tumor sizes, taken to be the longest dimension as measured and reported by Rapid Diagnostics Unit staff based on diagnostic ultrasound imaging, ranged from 0.5 cm to 4.4 cm with a median size of 2.3 cm. Examples of QUS parametric maps for representative malignant and benign cases are presented in Figure 3.

The RMSDs in mean primary QUS features and texture features for the malignant and benign patient cohorts are presented in Table 3 and Table 4, representing a comparison of root mean square feature estimate differences between ultrasound systems (USSs) to differences attributable to tissue heterogeneity. RMSD values were calculated separately for malignant (n = 22) and benign (n = 6) cohorts. The third column records the RMSD USS between the ST and L15 system data analyzed on their native −6 dB bandwidths, while the fourth column records the RMSD USS between system data analyzed on a common bandwidth. The average variation in estimated QUS and texture parameters due to breast tumor heterogeneity (RMSDf) was found to be 36% higher for the ST system than it was for CL15. Similarly, the mean variation due to heterogeneity for the benign patient cohort was found to be 85% higher for ST compared to CL15.

Since the RMSD USS calculated from breast masses is influenced by both hardware differences between the ultrasound systems and tissue heterogeneity, the average variation in QUS features and texture features estimated due to system differences (RMSD USS) was 2.7 and 1.7 times greater than the average variation attributable to tissue heterogeneity (RMSDf) for the malignant patient cohort, respectively. Similarly, for the benign patient cohort, the average variation in QUS features and texture features estimated due to system differences (RMSD USS) was 2.3 and 1.3 times greater than average variation attributable to tissue heterogeneity (RMSDf), respectively.

The ultrasound backscattering that occurs from a scatterer of a specific size depends on the frequency bandwidth of the ultrasound system for a specific transmit frequency. QUS and texture estimation from the ST and L15 data was performed for each set within their −6 dB frequency bandwidth. The variation in the QUS features and texture features estimated due to system differences (RMSD USS) was influenced mainly by differences in the frequency bandwidth of those two ultrasound systems. In order to investigate the actual variation in the estimated parameters due to the hardware of the ultrasound systems, the parameters were separately investigated over the same frequency bandwidth (5–8 MHz) for both ST and CL15 data. The RMSD USS calculated from QUS and texture parameters estimated for 5–8 MHz is included in Table 3 and Table 4. With the same frequency bandwidth, the variation in QUS parameters due to differences attributable to the ultrasound system was reduced to 55%.

Cross-system statistical significance testing of each of the 25 parameters was performed for the benign and malignant patient cohorts. The *p*-values are given in Table 5. The range of *p*-values was 1.03 × 10^−4^ to 0.827 for the benign cohort and 3.03 × 10^−10^ to 0.958 for the malignant cohort. For both cohorts, all five of the primary QUS features (MBF, SS, SI, ASD, AAC) were found to be in agreement at the 5% confidence level. A total of 13 of the 20 QUS texture features (65%) were determined to exhibit statistically significant differences in the sample medians of estimates between systems at the 5% confidence level, with the remaining 7 texture features being in agreement. The specific texture features found to be in agreement varied between benign and malignant cohorts; only mid-band fit contrast (MBF-con), spectral slope contrast (SS-con), and spectral intercept contrast (SI-con) were found to agree at the 5% level for both benign and malignant groups.

**Figure 4:**
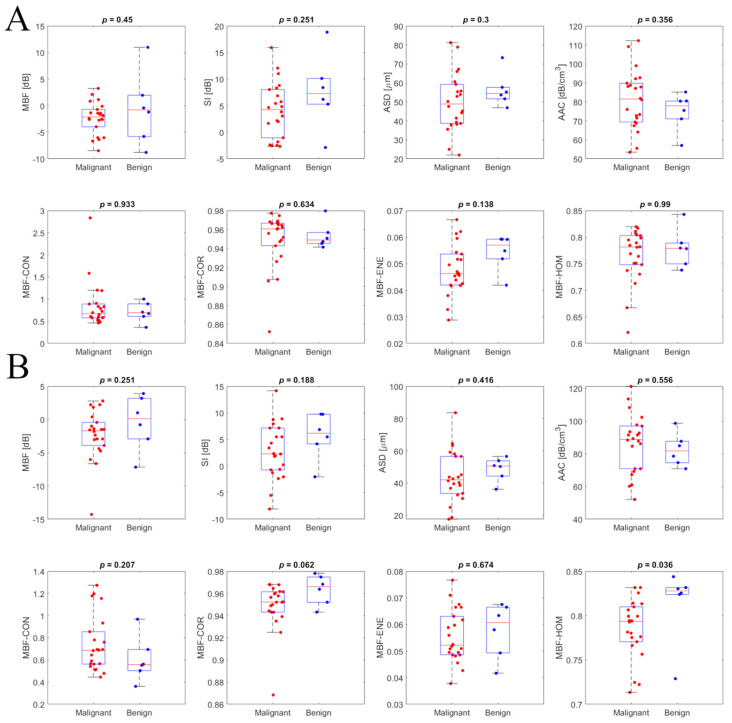
Intra-system comparison scatterplots for primary QUS features and texture features of the benign (in blue) and malignant (in red) patient groups for the Sonix Touch (**A**) and Clarius L15 HD (**B**) ultrasound systems.

Scatter plots for mean QUS and texture features of benign and malignant patient groups, for breast masses characterized using the ST and CL15 ultrasound systems, are presented in Figure 4. The backscatter intensity-related parameters, particularly MBF and SI calculated from ST and CL15 data, exhibited significant differences between malignant and benign groups. Figure 4 presents representative box plots of QUS features and texture features, separating the data into benign and malignant groups for the ST (top) and L15 (bottom) ultrasound systems. Statistical comparison using the Mann–Whitney U test was performed to assess the level of agreement between benign and malignant groups; *p*-values are included in the plots. In the box plot, the central red line indicates the median value, while the bottom and top edges represent the 25th and 75th percentiles, respectively.

**Figure 5:**
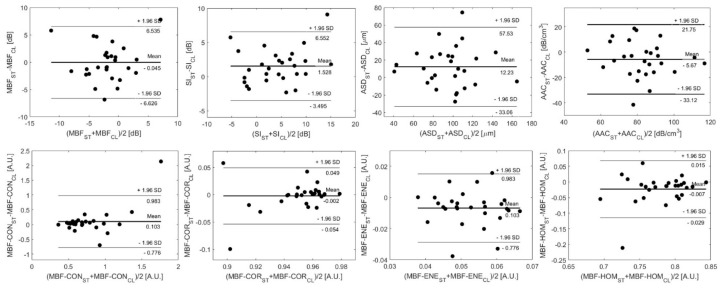
Bland–Altman plots comparing QUS and texture features acquired from breast lesions using the Sonix Touch (ST) and Clarius L15 HD (CL) ultrasound systems, plotting the difference in feature estimates between systems versus the inter-system mean estimate. The limits of agreement for feature estimates are ±1.96 standard deviations from the mean difference.

Bland–Altman plots for some representative features are displayed in Figure 5, with horizontal lines demarcating the mean difference and limits of agreement. The limits of agreement between parameters from the two clinical systems were defined by the mean difference ±1.96 standard deviations of the difference.

## 4. Discussion

Before analyzing the influence of ultrasound system hardware and transducer properties on QUS features, the effect of tissue heterogeneity on these parameters was investigated by analyzing the QUS and texture parameter estimates from images of breast lesions acquired using both a cart-based and a handheld ultrasound system. Differences in mean feature estimates attributable to tumor heterogeneity were found to be lower than those attributable to ultrasound system effects, but the magnitude of this difference was small. This finding indicates that variations in feature estimates attributable to differences in scanner properties for these two systems are comparable to differences attributable to factors such as tumor heterogeneity, varying tissue compression, and other influencing factors that can vary over the course of a single ultrasound scan.

The −6 dB frequency bandwidth of the ST system for a transmit–receive frequency of 10 MHz is wider (3–8 MHz) than the bandwidth of the CL15 system (5.1–8.3 MHz). For a frequency bandwidth range of 3–8 MHz, ultrasound backscatter occurs for scatterers sized from 80 to 200 µm. In the range of 5–8 MHz, ultrasound backscatter is influenced by scatterers sized from 80 to 125 µm. These differences in the scatterer size influence on ultrasound backscatter significantly impacted QUS feature estimates, especially for those features estimated from the BSC (ASD and AAC) and their corresponding texture parameter estimates.

For malignant breast tumors, the tissue heterogeneity of the tumor contributed significantly to the variation in feature estimates between frames of image data of the same tumor. Compared to texture parameters, variation in mean QUS values due to ultrasound system differences was higher, primarily because of the difference in the –6 dB frequency bandwidth range of the ultrasound transducers. To mitigate the variation in QUS resulting caused by system differences, the QUS and texture parameter estimates of the breast lesions acquired with Sonix Touch were analyzed in the 5.1–8.3 MHz frequency range and compared with the parameter estimates made using the L15 RF data. 

A significant decrease in the RMSDUS and no significant difference in mean primary QUS values between the two ultrasound scanners was observed. Agreement of texture feature estimates was poorer, with 13 of the 20 features investigated showing statistically significant differences for both benign and malignant groups. A previous study which compared QUS and texture parameter estimates between the Ultrasonix RP and the GE-LOGIQ E9 ultrasound scanners [12] reported that differences in beam properties including axial and lateral resolution have a greater impact on the texture features compared to the mean QUS parameters as demonstrated by lower *p*-values from statistical tests. The Clarius L15 HD system has an imaging resolution close to double that of the L14-5/60 transducer used with the Sonix Touch, and this large difference is one possible explanation for the significant disagreement between systems found for the texture feature estimates.

The Bland–Altman method (Figure 5) allowed for visual examination of the agreement between system feature estimates and confirmed the absence of any systematic errors introduced by the difference in measurement method. All five primary QUS features and the breast lesion patients’ parameters determined from the CL15 data were comparable with those estimated from the ST data. As explained in our previous study, for smaller lesions, there are fewer windows within the segmented ROI which results in fewer spatially mapped estimates of the features. Therefore, minor variations within the pixel levels related to transducer performance potentially result in larger differences in mean QUS and texture features between the two ultrasound systems with different beam properties. This was reflected in the Bland–Altman plots. The patients closer to the limits of the agreement (mean difference± 1.96 standard deviation) in the Bland–Altman plots are those with relatively smaller lesions, such as patients 3, 24, and 25.

A major limitation of the present study is the small size of the patient cohort under investigation (28 patients), and the size of the benign patient cohort in particular (6 patients). However, it should be noted that previous studies examining QUS feature estimates extracted from breast lesions have had similarly sized samples. Sannachi et al. [12], for example, compared feature estimates of locally advanced breast cancer between two clinical ultrasound scanners for a cohort of 24 breast patients, and Wirtzfeld et al. [11] compared BSC estimates of 4TI and MAT mammary tumors in animal models produced by different ultrasound systems for cohorts of 12 and 5 samples, respectively. 

Another limitation was the limited number of ultrasound device models under investigation; with only a single handheld and a single cart-based scanner with one model of transducer for each, the applicability of the results is limited to these specific systems and imaging settings. Expanding the diversity of transducer and system models under investigation in order to broadly verify the results is a likely avenue of future investigation.

In previous work, a breast lesion characterization model was developed based on quantitative ultrasound parameters, texture parameters, and texture derivative methods from a cohort of 208 patients whose image data were acquired with the Sonix Touch ultrasound system [1]. In this study, ultrasound backscatter intensity-related parameters indicated statistical significant difference between the benign and malignant groups. In particular, the mean values of MBF and SI were lower in the malignant group compared to benign patients. This observation is consistent with the observation from previous work where a breast lesion characterization model was developed based on a quantitative ultrasound texture derivative method involving 208 patients. These ultrasound backscatter intensity parameters are related to several tissue properties including scatterer size, shape, and elastic properties. Those properties differed between benign and malignant breast masses, as shown in histological analysis. In previous work, some of the texture features determined from the target regions demonstrated significant differences between the two groups. This is likely due to the smaller sample size in the benign group compared to our previous study.

## 5. Conclusions

Portable ultrasound systems offer several advantages over traditional cart-based systems, chief among these being their smaller form factor and the ease with which they can be transported to the point of care instead of having to bring the patient to a designated room for imaging. Their flexibility and relatively low cost compared to traditional scanners gives portable ultrasound the potential to dramatically increase the accessibility of ultrasound imaging and clinical QUS techniques, especially in markets such as developing countries where resources are limited. The main disadvantage of these systems at the time of writing is their typically lower imaging resolutions, but if it can be demonstrated that this limitation is not a barrier to using these portable systems in quantitative ultrasound applications, then they stand to offer a unique avenue of investigation. 

This study demonstrated that QUS feature estimates of breast masses made using both a portable ultrasound imaging system and a traditional cart-based ultrasound system were moderately comparable. Variation in feature estimates due to system differences were shown to be comparable in magnitude to the variation in feature estimates attributable to tumor tissue heterogeneity. The comparison of RMSD values between system feature estimates produced on each system’s native −6 dB bandwidth with estimates produced when the analysis was performed on the systems’ common bandwidth showed significantly increased agreement, underscoring the importance of selecting an appropriate analysis bandwidth when conducting comparisons of QUS results from different transducers. Statistical testing of feature estimates made by the two systems demonstrated good agreement for the primary QUS spectral and BSC-derived features, but relatively poor agreement between texture features derived from QUS feature parametric maps. We suggest that this discrepancy is likely attributable to the large difference in image resolution between systems. Bland–Altman analysis confirmed the absence of systematic errors in QUS feature estimates made using the two systems for both benign and malignant patient cohorts. 

A major limitation of the present study is the small overall sample size, and the size of the benign patient group in particular. Future studies will seek to examine a significantly larger patient population, as increased sample sizes will yield a more accurate picture of the level of agreement between different imaging systems and transducers and open up additional avenues for investigation. Future work will involve expanding the number of ultrasound systems under investigation, expanding the inter-system comparison to include a wider variety of manufacturers and transducer models and investigating the impact of effects such as inter-operator variability. Experimenting with methods for improving the agreement of texture feature estimates made by systems with large differences in image resolution is another potential direction of investigation that may have a large impact on improving the variety of ultrasound systems that can be used for performing QUS techniques. Assessing the viability of using feature estimates captured using a portable system as the input to previously developed multi-feature classification models trained using feature data acquired using cart-based ultrasound systems is another potential pathway of investigation. 

The methods employed in the present study can also be used to investigate the use of portable ultrasound for QUS feature estimation in other applications related to breast cancer, such as longitudinal studies of locally advanced breast cancer where QUS feature estimates are obtained at intervals over the course of a patient’s chemotherapy treatment and changes in feature estimates over time are tracked [6]. Clarius Mobile Health’s development of open-source software tools for research applications has also granted users more fine control of the Clarius L15HD’s imaging parameters; refining the scan settings used to collect ultrasound RF data could possibly improve the level of inter-system agreement of feature estimates.

## Figures and Tables

**Table 1:** Comparison of relevant ultrasound transducer and imaging parameters.

Parameters	ST (L14-5/60)	CL15
Transducer Parameters		
Number of Elements	128	192
Center Frequency [MHz]	6.3	6.7
Frequency Bandwidth range [MHz]	3–8	5.1–8.3
Imaging Parameters		
Sampling Rate [MHz]	40	30
Focal Position [mm]	17.5	20.03
Image Pixel Width (Lateral) [mm]	0.1174	0.2604
Image Pixel Width (Axial) [mm]	0.0193	0.0256
Image Size (Lateral) [pixels]	510	192
Image Size (Axial) [pixels]	2064	1586

**Table 2:** Summary of patient characteristics.

ID	Benign/Malignant	Mass Size (cm)	Age	Side	Grade	Histology	ER	PR	HER2
1	M	4.4	52	L	2	Invasive Lobular Carcinoma (ILC)	+	+	-
2	M	2.1	49	L	1	Invasive Ductal Carcinoma (IDC)	+	+	-
3	M	1.0	65	R	1	IDC	+	+	-
4	M	2.6	41	L	2	IDC	+	+	-
5	M	4.1	97	L	2	Metaplastic Breast Carcinoma	-	-	-
6	B	2.4	39	L	N/A	Fibroadenoma (Benign)	N/A	N/A	N/A
7	M	2.2	78	R	1	IDC	+	+	-
8	B	3.4	34	L	N/A	Fibroadenoma (Benign)	N/A	N/A	N/A
9	B	1.5	44	R	N/A	Fibroadenoma (Benign)	N/A	N/A	N/A
10	M	5.9	84	L	1	ILC	+	-	-
11	B	1.2	46	L	N/A	Fibroepithelial Lesion (Benign)	N/A	N/A	N/A
12	M	2.0	54	R	2	Mixed IDC/ILC	+	+	-
13	M	4.4	60	R	2	ILC	+	+	-
14	M	3.4	44	L	3	IDC	-	-	+
15	M	2.5	56	R	3	IDC	-	-	-
16	M	2.6	46	L	3	IDC	-	-	-
17	M	1.7	53	R	2	IDC	+	+	-
18	M	3.8	52	L	2	IDC	+	-	+
19	M	1.9	52	L	2	IDC	-	-	-
20	M	3.4	53	L	2	IDC	+	+	+
21	M	2.2	39	L	2	IDC	+	+	+
22	M	1.1	35	L	1	IDC	+	+	-
23	B	2.9	38	R	N/A	Fibroadenoma (Benign)	N/A	N/A	N/A
24	M	0.5	50	L	2	IDC	+	+	-
25	B	0.7	55	L	N/A	Microcysts with Calcifications (Benign)	N/A	N/A	N/A
26	M	3.6	62	R	2	Mixed IDC/ILC	+	+	-
27	M	1.2	43	R	2	IDC, Micropapillary Features	+	+	-
28	M	1.4	48	R	2	IDC	+	+	-

**Table 3:** Results of QUS consistency analysis for malignant patient group.

QUS Feature	RMSD (ST) (3–8 MHz)	RMSD (L15) (5–8 MHz)	RMSD USS ST (3–8 MHz) and L15 (5–8 MHz)	RMSD USS (5–8 MHz)
ASD	16.34	8.951	34.82	13.658
AAC	17.71	12.084	35.95	16.072
MBF	3.237	2.706	3.48	3.048
SS	0.509	0.3975	1.31	0.531
SI	1.815	2.206	7.62	2.574
ASD-CON	0.323	0.264	0.381	0.542
ASD-COR	0.0230	0.0147	0.0237	0.028
ASD-ENE	0.0380	0.0420	0.0864	0.041
ASD-HOM	0.0336	0.0257	0.0650	0.083
AAC-CON	0.539	0.374	0.756	0.969
AAC-COR	0.0302	0.0146	0.0342	0.035
AAC-ENE	0.0557	0.0188	0.0879	0.022
AAC-HOM	0.0515	0.0267	0.0630	0.088
MBF-CON	0.178	0.141	0.302	0.492
MBF-COR	0.0138	0.0104	0.0239	0.028
MBF-ENE	0.0109	0.00970	0.0125	0.014
MBF-HOM	0.0236	0.0209	0.0585	0.052
SS-CON	0.148	0.155	0.182	0.229
SS-COR	0.0206	0.0127	0.0162	0.025
SS-ENE	0.00977	0.0136	0.0141	0.008
SS-HOM	0.0189	0.0175	0.0363	0.036
SI-CON	0.156	0.122	0.228	0.274
SI-COR	0.0159	0.0125	0.0152	0.026
SI-ENE	0.0104	0.0104	0.0152	0.009
SI-HOM	0.0188	0.0147	0.0402	0.036

**Table 4:** Results of QUS consistency analysis for benign patient group, as per Table 3.

QUS Feature	RMSD (ST) (3–8 MHz)	RMSD (L15) (5–8 MHz)	RMSD USS ST (3–8 MHz) and L15 (5–8 MHz)	RMSD USS (5–8 MHz)
ASD	21.25	12.02	21.53	8.514
AAC	21.44	13.61	15.69	7.074
MBF	3.052	2.35	7.132	4.262
SS	0.622	0.409	0.816	0.296
SI	2.152	2.383	10.34	4.253
ASD-CON	0.269	0.204	0.205	0.487
ASD-COR	0.0150	0.0153	0.0183	0.025
ASD-ENE	0.0281	0.0125	0.0327	0.019
ASD-HOM	0.0293	0.0181	0.0343	0.075
AAC-CON	0.474	0.282	0.484	0.792
AAC-COR	0.0224	0.0152	0.0284	0.030
AAC-ENE	0.0539	0.0180	0.0621	0.021
AAC-HOM	0.0529	0.0241	0.0322	0.082
MBF-CON	0.145	0.138	0.136	0.172
MBF-COR	0.129	0.00964	0.0186	0.017
MBF-ENE	0.0158	0.0111	0.0117	0.006
MBF-HOM	0.0284	0.0195	0.0456	0.049
SS-CON	0.0978	0.147	0.125	0.186
SS-COR	0.0169	0.0139	0.0179	0.026
SS-ENE	0.00867	0.00840	0.0129	0.011
SS-HOM	0.0159	0.0193	0.0239	0.048
SI-CON	0.150	0.189	0.119	0.125
SI-COR	0.0129	0.0134	0.0158	0.016
SI-ENE	0.00947	0.0111	0.0149	0.013

**Table 5:** Results of QUS consistency analysis for benign patient group.

QUS Feature	*p*-Value, Benign Group	*p*-Value, Malig. Group
MBF	0.827	0.958
SS	0.182	0.165
SI	0.543	0.254
ASD	0.228	0.171
AAC	0.661	0.211
MBF-con	0.0995	0.385
MBF-cor	0.0430	0.940
MBF-ene	0.372	0.0140
MBF-hom	0.00389	0.159
SS-con	0.209	0.0609
SS-cor	0.0518	0.00664
SS-ene	0.0361	0.142
SS-hom	0.00451	0.00249
SI-con	0.273	0.0738
SI-cor	0.203	0.0455
SI-ene	0.0164	0.0656
SI-hom	0.00230	0.00344
ASD-con	0.0167	0.00127
ASD-cor	0.0436	0.00619
ASD-ene	0.278	0.0216
ASD-hom	6.58 × 10^−4^	1.02 × 10^−9^
AAC-con	0.0102	7.87 × 10^−4^
AAC-cor	0.0388	0.00393
AAC-ene	0.192	0.0135
AAC-hom	1.03 × 10^−4^	3.03 × 10^−10^

## Data Availability

The data presented in this study are available on request from the corresponding author in accordance with the institutional policies of Sunnybrook Research Institute.

## References

[1] Osapoetra L.O., Sannachi L., DiCenzo D., Quiaoit K., Fatima K., Czarnota G.J. (2020). Breast lesion characterization using Quantitative Ultrasound (QUS) and derivative texture methods. Transl. Oncol..

[2] Oh K.E., Vasandani N., Anwar A. (2023). Radiomics to Differentiate Malignant and Benign Breast Lesions: A Systematic Review and Diagnostic Test Accuracy Meta-Analysis. Cureus.

[3] Sadeghi-Naini A., Falou O., Tadayyon H., Al-Mahrouki A., Tran W., Papanicolau N., Kolios M.C., Czarnota G.J. (2013). Conventional Frequency Ultrasonic Biomarkers of Cancer Treatment Response In Vivo. Transl. Oncol..

[4] Sadeghi-Naini A., Sannachi L., Pritchard K., Trudeau M., Gandhi S., Wright F.C., Zubovits J., Yaffe M.J., Kolios M.C., Czarnota G.J. (2014). Early prediction of therapy responses and outcomes in breast cancer patients using quantitative ultrasound spectral texture. Oncotarget.

[5] Sadeghi-Naini A., Suraweera H., Tran W.T., Hadizad F., Bruni G., Rastegar R.F., Curpen B., Czarnota G.J. (2017). Breast-Lesion Characterization using Textural Features of Quantitative Ultrasound Parametric Maps. Sci. Rep..

[6] DiCenzo D., Quiaoit K., Fatima K., Bhardwaj D., Sannachi L., Gangeh M., Sadeghi-Naini A., Dasgupta A., Kolios M.C., Trudeau M. (2020). Quantitative ultrasound radiomics in predicting response to neoadjuvant chemotherapy in patients with locally advanced breast cancer: Results from multi-institutional study. Cancer Med..

[7] Nam K., Rosado-Mendez I.M., Wirtzfeld L.A., Kumar V., Madsen E.L., Ghoshal G., Pawlicki A.D., Oelze M.L., Lavarello R.J., Bigelow T.A. (2012). Cross-imaging system comparison of backscatter coefficient estimates from a tissue-mimicking material. J. Acoust. Soc. Am..

[8] Anderson J.J., Herd M.-T., King M.R., Haak A., Hafez Z.T., Song J., Oelze M.L., Madsen E.L., Zagzebski J.A., O’Brien W.D. (2010). Interlaboratory Comparison of Backscatter Coefficient Estimates for Tissue-Mimicking Phantoms. Ultrason. Imaging.

[9] Nam K., Rosado-Mendez I.M., Wirtzfeld L.A., Pawlicki A.D., Kumar V., Madsen E.L., Ghoshal G., Lavarello R.J., Oelze M.L., Bigelow T.A. (2011). Ultrasonic Attenuation and Backscatter Coefficient Estimates of Rodent-Tumor-Mimicking Structures: Comparison of Results among Clinical Scanners. Ultrason. Imaging.

[10] Wirtzfeld L.A., Ghoshal G., Hafez Z.T., Nam K., Labyed Y., Anderson J.J., Herd M.-T., Haak A., He Z., Miller R.J. (2010). Cross-Imaging Platform Comparison of Ultrasonic Backscatter Coefficient Measurements of Live Rat Tumors. J. Ultrasound Med..

[11] Wirtzfeld L.A., Ghoshal G., Rosado-Mendez I.M., Nam K., Park Y., Pawlicki A.D., Miller R.J., Simpson D.G., Zagzebski J.A., Oelze M.L. (2015). Quantitative Ultrasound Comparison of MAT and 4T1 Mammary Tumors in Mice and Rats Across Multiple Imaging Systems. J. Ultrasound Med..

[12] Sannachi L., Gangeh M., Naini A.-S., Bhargava P., Jain A., Tran W.T., Czarnota G.J. (2020). Quantitative Ultrasound Monitoring of Breast Tumour Response to Neoadjuvant Chemotherapy: Comparison of Results Among Clinical Scanners. Ultrasound Med. Biol..

[13] Nelson B.P., Topol E., Bhagra A., Mulvagh S.L., Narula J. (2018). Atlas of Handheld Ultrasound.

[14] Le M.-P.T., Voigt L., Nathanson R., Maw A.M., Johnson G., Dancel R., Mathews B., Moreira A., Sauthoff H., Gelabert C. (2022). Comparison of four handheld point-of-care ultrasound devices by expert users. Ultrasound J..

[15] Moussaoui G., Zakaria A.S., Negrean C., Nguyen D.-D., Couture F., Tholomier C., Sadri I., Arezki A., Schwartz R.N., Elterman D.S. (2021). Accuracy of Clarius, Handheld Wireless Point-of-Care Ultrasound, in Evaluating Prostate Morphology and Volume Compared to Radical Prostatectomy Specimen Weight: Is There a Difference between Transabdominal *vs* Transrectal Approach?. J. Endourol..

[16] Insana M.F., Hall T.J. (1990). Parametric ultrasound imaging from backscatter coefficient measurements: Image formation and interpretation. Ultrasonic Imaging..

[17] Insana M.F., Wagner R.F., Brown D.G., Hall T.J. (1990). Describing small-scale structure in random media using pulse-echo ultrasound. J. Acoust. Soc. Am..

[18] Yao L.X., Zagzebski J.A., Madsen E.L. (1990). Backscatter Coefficient Measurements Using a Reference Phantom to Extract Depth-Dependent Instrumentation Factors. Ultrason Imaging.

[19] Madsen E.L., Zagzebski J.A., Frank G.R. (1982). Oil-in-gelatin dispersions for use as ultrasonically tissue-mimicking materials. Ultrasound Med. Biol..

[20] Kremkau F.W., Barnes R.W., McGraw C.P. (1981). Ultrasonic attenuation and propagation speed in normal human brain. J. Acoust. Soc. Am..

[21] Duric N., Littrup P., Babkin A., Chambers D., Azevedo S., Kalinin A., Pevzner R., Tokarev M., Holsapple E., Rama O. (2005). Development of ultrasound tomography for breast imaging: Technical assessment. Med. Phys..

[22] Labyed Y., Bigelow T.A. (2011). A theoretical comparison of attenuation measurement techniques from backscattered ultrasound echoes. J. Acoust. Soc. Am..

[23] Oelze M.L., O’Brien W.D. (2002). Method of improved scatterer size estimation and application to parametric imaging using ultrasound. J. Acoust. Soc. Am..

[24] Gerig A., Zagzebski J., Varghese T. (2003). Statistics of ultrasonic scatterer size estimation with a reference phantom. J. Acoust. Soc. Am..

[25] Li C., Duric N., Littrup P., Huang L. (2009). In vivo Breast Sound-Speed Imaging with Ultrasound Tomography. Ultrasound Med. Biol..

[26] Haralick R.M., Shanmugam K., Dinstein I. (1973). Textural Features for Image Classification. IEEE Trans. Syst. Man Cybern. SMC-.

[27] Bland J.M., Altman D.G. (1986). Statistical methods for assessing agreement between two methods of clinical measurement. Lancet.

